# Self-confidence for emergency intervention: adaptation and cultural validation of the Self-confidence Scale in nursing students

**DOI:** 10.1590/0104-1169.3128.2451

**Published:** 2014-07-22

**Authors:** José Carlos Amado Martins, Rui Carlos Negrão Baptista, Verónica Rita Dias Coutinho, Alessandra Mazzo, Manuel Alves Rodrigues, Isabel Amélia Costa Mendes

**Affiliations:** 1 Post-doctoral fellow, Escola de Enfermagem de Ribeirão Preto, Universidade de São Paulo, WHO Collaborating Centre for Nursing Research Development, Ribeirão Preto, SP, Brazil. Scholarship holder from Fundação para a Ciência e Tecnologia, Portugal. Coordinator Professor, Escola Superior de Enfermagem de Coimbra, Coimbra, Portugal; 2 Doctoral student, Instituto de Ciências Biomédicas, Universidade do Porto, Porto, Portugal and Escola Superior de Enfermagem do Porto, Porto, Portugal. Adjunct Professor, Escola Superior de Enfermagem de Coimbra, Coimbra, Portugal; 3 PhD, Professor, Escola de Enfermagem de Ribeirão Preto, Universidade de São Paulo, WHO Collaborating Centre for Nursing Research Development, Ribeirão Preto, SP, Brazil; 4 PhD, Coordinator Professor, Escola Superior de Enfermagem de Coimbra, Coimbra, Portugal; 5 PhD, Full Professor, Escola de Enfermagem de Ribeirão Preto, Universidade de São Paulo, WHO Collaborating Centre for Nursing Research Development, Ribeirão Preto, SP, Brazil

**Keywords:** Self-Confidence, Patient Simulation, Emergency Nursing

## Abstract

**Objective:**

develop the cultural adaptation and validation of a Portuguese version of the Self-confidence Scale.

**Method:**

descriptive and exploratory methodological research for the adaptation and validation of a measuring instrument. The translation, synthesis, back-translation, revision, pretest and semantic evaluation phases were accomplished. The evaluation involving 178 students from a Teaching Diploma Program in Nursing. The ethical principles were complied with.

**Results:**

the internal consistency analysis of the scale reveals good Alpha coefficients (0.92 for the global scale and superior to 0.83 for the different dimensions). The factor analysis presents a three-factor solution with rational meaning.

**Conclusion:**

The scale is easy to answer and understand. Based on the obtained results, it can be affirmed that the scale reveals good psychometric properties, with great potential to be used in future research.

## Introduction

In an emergency situation, one of the factors that can influence the rapid and appropriate initiation of the nursing intervention is the self-confidence of the professional present when this intervention takes place. The organization of care turns the nurse into the core element of this process.

When nurses graduate, they are expected to have gained the knowledge and competences, among others, to identify signs and symptoms, to assess the patient in a fast and systematic manner, to implement interventions according to their degree of priority and to evaluate the outcome of the interventions. That is the only way to achieve high quality in the intervention response to complex situations like a stroke, which can double or triple the patient's survival^(^ ^1^ ^)^.

Therefore, students' education depends not only on the cases they are confronted with during the different clinical teaching periods. Schools are expected to reinvent themselves, using innovative pedagogical strategies that develop competences in the students, which allow them to act in high-complexity contexts, in which decision making is based on scientific evidence and derives from an easy, structured and fluid clinical judgment, with high levels of self-confidence. The pedagogical strategies need to facilitate the integrative construction of knowledge, as well as reflexive observation and experiencing through immersion in the reality^(^ ^2^ ^)^, so as to transmit security to the different actors^(^ ^3^ ^)^.

When included into the study plans of nursing colleges, simulation teaching is one way to achieve this competency building. A good study plan, which includes simulation, can contribute to the preparation of better nurses, who are capable of intervening in complex situations, of making correct decisions centered on each person and based on scientific evidence, of working in teams and of actively working to update their knowledge and competences, among others. In other words, it prepares people who know (considering knowledge in its different dimensions), who know what they know and who know everything they can still learn.

It should be observed that the replacement of clinical experience by simulated clinical experience is not defended here. Contact with real persons permits singular learning. The main advantage simulated practice has to offer is a safe environment for the student, the teacher and the patient in the teaching and learning process, in addition to its power to anticipate rare and/or complex situations^(^ ^3^ ^)^.

In this context, the assessment of self-confidence to intervene in an urgency situation can be very useful. In view of the above, the objective in this study was to develop the cultural adaptation and validation of a Portuguese version of the Self-confidence Scale.

Self-confidence, also described as self-efficacy, is always related to a behavior or task^(^ ^4^ ^)^. Confidence is an attitude, frequently related to repeated experiences and to the realistic perception of individual weaknesses and potentials.

Confidence is an important variable in nursing education^(^ ^5^ ^)^. Students with higher levels of self-confidence have a greater probability of developing successful interventions, as they are able to test and use their competences more easily^(^ ^5^ ^)^. Despite appropriate knowledge and skills, nurses are generally reluctant to start certain interventions, unless they feel confident to do so^(^ ^6^ ^)^.

According to the theory of self-efficacy^(^ ^4^ ^)^, individuals with a greater sense of self-efficacy or self-confidence are more willing to accept challenges and recover faster from failure^(^ ^5^ ^)^.

The development of self-confidence is the main component of correct decision making in the clinical context and for the associated judgment processes^(^ ^7^ ^)^. Various studies show that self-confidence to react to emergency situations increases when factors like repeated practice^(^ ^5^ ^)^ and simulation training^(^ ^3^ ^,^ ^5^ ^,^ ^8^ ^-^ ^15^ ^)^ are present.

In fact, the use of simulation as a teaching/learning strategy reveals different gains for the students, particularly the development of knowledge and competences for clinical judgment, priority setting, decision making, accomplishment of correct actions, teamwork and correction of errors without impairing the patients; and, associated with all these, gains in self-confidence levels^(^ ^3^ ^)^. Other studies^(^ ^16^ ^-^ ^17^ ^)^, centered on outcomes related to the use of high-fidelity simulation, also indicate gains in the level of self-confidence.

In emergency interventions, studies also demonstrate the importance of self-confidence. Examples are the relation between self-confidence and nurses' ability to recognize and appropriately respond to an emergency situation^(^ ^7^ ^)^ or, in trauma emergency interventions, the higher self-confidence levels of students submitted to simulation training when compared to others who only participate in seminar training^(^ ^8^ ^)^. Another example is how a structured simulation training program improves the students' confidence to identify signs of worsening in a patient^(^ ^18^ ^)^.

The analysis of the most common errors during the cardiorespiratory reanimation of a patient and its causes shows that, in different nurses, errors are related to high levels of anxiety and low levels of self-confidence^(^ ^19^ ^)^.

A study shows that the relation between self-confidence and the result of emergency interventions can be transferred to practice^(^ ^11^ ^)^.

No scientific evidence exists yet, however, regarding the effectiveness of simulated practice. It knowingly contributes to increase self-confidence levels when compared to mere theoretical education, but there are no statistically significant differences when compared to clinical practice^(^ ^5^ ^)^. In the same study^(^ ^5^ ^)^, simulated practice did not significantly contribute to greater knowledge retention and clinical performance. The combination of clinical practice and simulation obtained better outcomes than any of the two individually.

## Objective

To develop the cultural adaptation and validation of a Portuguese version of the Self-confidence Scale.

## Method

Methodological adaptation and validation study of a measuring instrument. For the study, a minimum of 120 participants was estimated, attending to the target of 10 participants for each scale item^(^ ^20^ ^)^. The sample consists of 178 students (56% of the population) who complied with the inclusion criteria: having participated in the practical classes on Emergency Nursing and accepting to participate in the research voluntarily. More than three quarters (76.8%) of the participants were women. The mean and median age was 22 years and the mode 21 years, ranging between 20 and 32 years. The standard deviation corresponds to 1.9 years. Most students (59.1%) participated actively in the simulated clinical experience with a scenario developed in a high-fidelity simulator. The remainder participated in two (19.9%), three (5.5%) or more (5.1%) simulated clinical experiences (19 students did not answer this question).

According to the students' answers, the average time needed to answer the SCS^vp^ was three minutes and a half.

The Self-confidence Scale (SCS) was developed by Frank Hicks in 2006 and was published in the work by Hicks, Coke and Li in 2009^(^ ^5^ ^)^. The central objective in the development of the SCS was to evaluate the self-confidence variable, subdivided in four dimensions. The scale consists of a list of 12 items with five-point Likert answers: "not confident", "hardly confident", "confident", "very confident" and "extremely confident". The different items identify the student's ability to: (1) recognize signs and symptoms of changes in the referred areas, (2) assess the patient precisely, (3) intervene appropriately and (4) assess the effectiveness of the interventions implemented in the respiratory, cardiac and neurological areas.

To adapt the instrument, the following phases were undertaken^(^ ^21^ ^)^:

- Translation of the instrument to Portuguese by three of the authors, who were fluent in English, one of whom with profound theoretical and clinical knowledge on emergency interventions;
- Synthesis of the translation to a single document;
- Revision by two experts in English/Portuguese, who did not make any changes;
- Back-translation by experts in English/Portuguese (conceptual and semantic agreement levels close to 100.0%);
- Revision of translations by expert panel (the researchers and four nurses working in emergency care at a Portuguese university hospital, who are experienced in college teaching and projects). Given the simplicity of the instrument and clear formulation of the sentences, the conceptual, cultural, semantic and idiomatic agreement practically corresponded to 100.0%.
- Formatting to the original layout, maintaining the five response hypotheses;
- Pre-test. The pre-test involved 45 students in January 2011 to verify the participants' understanding of the questionnaire. No changes were necessary. The average time needed to answer the questionnaire was four minutes. The initials *vp* (*versão portuguesa*) were added to the Portuguese version.

For the purpose of the study, the minimum number of participants was estimated at 120, complying with the target of 10 participants for each scale item^(^ ^21^ ^)^.

The data were collected through a questionnaire, applied in April 2011 to fourth-year students from the Teaching Diploma Program in Nursing offered at the *Escola Superior de Enfermagem de Coimbra* (ESEnfC), after 18 hours of laboratory practice, using simulation, as part of the curricular unit Emergency Nursing.

The practical classes took place at the Simulation Center, during three-hour periods per week, and used the solution of complete scenarios in a realistic setting as a strategy, with increasing levels of difficulty. During each period, on average, the students participated in six scenarios (actively or as observers): two pediatric scenarios (one with a high and one with a medium-fidelity simulator) and four adult scenarios (two with a high and one with a medium-fidelity simulator). To solve the scenarios, the students had realistic materials and equipment at their disposal. Medium-fidelity (Megacod^(r)^ adult and junior advanced life-support manikins, with VitalSim^(r)^, by Laerdal^(r))^ and high-fidelity patient simulators were used (iStan^(r)^ (adult) and PediaSim^(r)^ (Junior) by Meti^(r))^.

All students enrolled for the curricular unit participated in the classes, totaling 318. In this course phase, the students had not participated in a practicum at an emergency service yet, but might have had some contact with critical patients at the other clinics used for teaching practicums.

Authorization was obtained from the author of the original SCS version, Dr. Frank Hicks.

The research is part of the Project "Simulation in Nursing Teaching", undertaken by the Health Science Research Unit - Nursing at ESEnfC. The project was assessed by the Ethics Committee of the same Research Unit, receiving a positive opinion (P 01-09/2010), and was authorized by the School President.

The students personally received explanations about the research objectives and the voluntary nature of their participation. They answered the scale after the formal evaluation of the curricular unit, guarantee that the scale answers would not be related with the students' assessment. A written informed consent form was used.

### Data analysis

The data were analyzed using SPSS software (version 19 for Windows). For all tests, statistical significance was set at *p*<0.05.

The answers to the different items were scored between 1 ("not confident") and 5 ("extremely confident").

## Results

The matrix revealed correlation coefficients between 0.35 and 0.80, with *p*<0.001. A high correlation was found between practically all items and the total scale, showing its good functioning as a whole and contributing to the high Alpha coefficient (0.918). In addition, all of the items contribute to a good Alpha coefficient, showing that the elimination of any of them would negatively affect the scale.

**Table 1:** Homogeneity statistics of the items and Cronbach's internal consistency coefficients of the SCSvp (N=178). Coimbra, Portugal, 2011

Items	Mean	Standard Deviation	Correlation with the total (corrected)	Alpha if the item were eliminated
1	3.34	0.55	0.56	0.915
2	3.56	0.54	0.58	0.914
3	3.02	0.67	0.63	0.912
4	3.32	0.67	0.69	0.909
5	3.63	0.62	0.69	0.910
6	3.23	0.76	0.64	0.913
7	3.17	0.68	0.70	0.909
8	3.56	0.63	0.69	0.910
9	3.06	0.69	0.70	0.909
10	3.24	0.61	0.67	0.910
11	3.48	0.63	0.70	0.909
12	4.19	0.65	0.71	0.909

Cronbach's Alpha (32 items): 0.964

The high internal consistency coefficients served to stimulate the construct analysis. Although the original version of the scale was rationally divided in four dimensions, the researchers attempted to analyze the appropriateness of this division for the Portuguese population, using factor analysis with orthogonal Varimax rotation with the Kaiser normalization^(^ ^22^ ^-^ ^23^ ^)^.

As a suitability criterion of the factor analysis, the *Kaiser-Meyer Olkin *(KMO) tests were used (0.867), whose value recommends the factor analysis and Bartlett's sphericity test (χ^2^=1390.084 with *p*<0.001), whose value shows that the variables can be related^(^ ^22^ ^)^. The presented solution proposed the division of the items in three factors, which in total explain 71.4% of the variance. The factor loadings of the three factors can be observed in Table 2.

**Table 2:** Saturation matrix of the items in the factors for the orthogonal Varimax rotated solution with Kaiser normalization (N=178). Coimbra, Portugal, 2011

Items		Factor 1	Factor 2	Factor 3
1.	*Quão confiante está de ser capaz de reconhecer sinais e sintomas de um evento cardíaco?*		0.322	0.594
2.	*Quão confiante está de ser capaz de reconhecer sinais e sintomas de um evento respiratório?*	0.428	0.575	
3.	*Quão confiante está de ser capaz de reconhecer sinais e sintomas de um evento neurológico?*	0.817		
4.	*Quão confiante está de ser capaz de avaliar com precisão um indivíduo com dor torácica?*			0.746
5.	*Quão confiante está de ser capaz de avaliar com precisão um indivíduo com dispneia?*		0.833	
6.	*Quão confiante está de ser capaz de avaliar com precisão um indivíduo com alteração do estado mental?*	0.759		
7.	*Quão confiante está de ser capaz de intervir apropriadamente num indivíduo com dor torácica?*			0.845
8.	*Quão confiante está de ser capaz de intervir apropriadamente num indivíduo com dispneia?*		0.834	0.307
9.	*Quão confiante está de ser capaz de intervir apropriadamente num indivíduo com alteração do estado mental?*	0.763		
10.	*Quão confiante está de ser capaz de avaliar a eficácia das suas intervenções num indivíduo com dor torácica?*			0.870
11.	*Quão confiante está de ser capaz de avaliar a eficácia das suas intervenções num indivíduo com dispneia?*		0.732	0.383
12.	*Quão confiante está de ser capaz de avaliar a eficácia das suas intervenções num indivíduo com alteração do estado mental?*	0.705		

Note : Eigenvalues below 0.30 were omitted

The analysis of the item distribution showed that the mathematically proposed grouping divides the scale in three dimensions, with items focused on breathing, circulation and neurological dysfunction. Although the proposed solution differs from the original scale author's proposal, it also has a rational meaning, by dividing the items into the breathing, cardiac and neurological areas and grouping the identification of signs and symptoms of severity, the patient assessment, the intervention and the assessment of the outcomes associated with the intervention in each of these dimensions.

Thus, the SCSvp consists of three factors. Factor 1, which includes the items related to the "neurological dysfunction" dimension of the scale; factor 2, which includes the items related to the "breathing" dimension of the scale; and factor 3, which includes the items related to the "circulation" dimension of the scale. Table 3 presents the alpha coefficients for each of the dimensions, showing that, despite the small number of items, the alpha coefficients are high.

**Table 3:** Alpha coefficient for each of the factors and for the global SCSvp (N=178). Coimbra, Portugal, 2011

Factor (dimension)	No. of items	Cronbach’s Alpha
Factor 1 (neurological dysfunction)	4	0.866
Factor 2 (respiratory)	4	0.858
Factor 3 (cardiac)	4	0.836
Global SCS*vp*	12	0.918

The variance explained by the three factors corresponds to 26.8%, 23.1% and 21.4%, respectively.

The mutual correlations between these three factors and with the global scale is high and very significant in statistical terms, as observed in the results displayed in Table 4.

**Table 4:** Pearson correlation matrix between factors and with the global SCSvp (N=178). Coimbra, Portugal, 2011

	Factor 1			Factor 2			Factor 3			Factor 4	
	r	p		r	p		r	p		r	p
Factor 1 (neurological dysfunction)				0.602	0.000		0.627	0.000		0.868	0.000
Factor 2 (respiratory)	0.602	0.000					0.679	0.000		0.863	0.000
Factor 3 (cardiac)	0.627	0.000		0.679	0.000					0.877	0.000
Global SCS*vp*	0.868	0.000		0.863	0.000		0.877	0.000			

In Table 5, the descriptive statistical results for the global scale and each of its factors are displayed. Globally, the students are self-confident, even if modestly, about their abilities to intervene in an emergency situation. Ranging between one and five, the mean coefficient corresponds to 3.32 with a slightly higher median. In combination with the percentiles, this means that the answers mostly varied between "confident" and "very confident". The dimension with the best evaluations was related to care for the patient's breathing in emergency situations, while the lowest scores were related to neurological dysfunctions in an emergency situation.

**Table 5:** Descriptive statistics of factors and global SCSvp (N=178). Portugal, 2011

SCS*vp* Statistics		Factor 1 (neurological dysfunction)	Factor 2 (respiratory)	Factor 3 (cardiac)	Global SCSvp
Mean		3.12	3.55	3.26	3.32
Median		3.00	3.50	3.25	3.33
Mode		3.00	4.00	3.00	3.00
Standard Deviation		0.59	0.51	5.28	0.47
Variance		0.34	0.26	0.28	0.22
Minimum		1.50	2.00	2.00	2.00
Maximum		5.00	5.00	4.75	4.50
Percentiles					
	25	2.75	3.00	3.00	3.00
	50	3.00	3.50	3.25	3.33
	75	3.50	4.00	3.75	3.67

## Discussion

The results found are promising with regard to the potential use of the SCS*vp*. One of the strengths is the simplicity and clarity of the items and their relation with the main intervention priorities in emergency situations. The scale is easy and fast to answer, and its items cover the main aspects for a high-quality intervention in an emergency situation. This simplicity facilitated the translation and content and semantic validity process.

The high internal consistency coefficients are a good indicator of the scale's behavior as a whole. The coefficients found border on the original study^(^ ^5^ ^)^, in which the Alpha coefficients on the pre and post-test corresponded to 0.93 and 0.96, respectively.

In the original scale, the author rationally divided the items into four dimensions, related to the identification of signs and symptoms, the patient's assessment, the intervention and the assessment of the interventions' effectiveness. When we try to mathematically divide the scale through factor analysis, the proposed solution divides the scale in three dimensions that explain 71.9% of the variance. The rational meaning of the solution proposed by the factor analysis is a good indicator^(^ ^21^ ^)^ that underlines the construct validity of the scale construct. The clear saturation of the items in one of the factors is a good indicator of convergent-discriminant validity, as it suggests that the item measures the same construct as the dimension it belongs to instead of any other^(^ ^21^ ^)^.

The high Alpha coefficients in each of the dimensions (superior to 0.83) and the high mutual correlations among the dimensions and with the global scale also indicate the solidity of the construct.

The descriptive analysis results show above-average self-confidence levels in the different dimensions and globally, with higher self-confidence rates in the dimension "breathing" and lower rates in the "neurological dysfunction" dimensions. These differences are explained by the degree of difficulty to assess and intervene in each of the dimensions and also serve as a positive indicator of the dimensions' distinguishing nature.

## Conclusions

The self-confidence to intervene in an emergency situation indicates the nurses' pro-activeness. To intervene in an emergency situation, in which each second matters, it is fundamental for nurses to feel confident that they are capable of acting appropriately, while low self-confidence can take the form of delayed help, higher levels of anxiety and more errors.

Simulated practice is a strategy that can enhance the self-confidence to intervene in an emergency situation.

The Self-confidence Scale showed good psychometric properties, which reveals the high potential use of the SCS*vp*, whether in research or as a tool to assess the quality of the trainers' work in simulated training contexts.

The limitations, such as the specific nature of the sample and the (also specific) context need to be considered in future studies, as well as the lack of concurrent validity and test-retest analyses in order to consolidate the validity of the SCS*vp*, as well as to strengthen its potential use.
